# Recombinant PNPLA1 catalyzes the synthesis of acylceramides and acyl acids with selective incorporation of linoleic acid

**DOI:** 10.1016/j.jlr.2023.100379

**Published:** 2023-04-21

**Authors:** Jason M. Meyer, William E. Boeglin, Alan R. Brash

**Affiliations:** 1 Department of Dermatology, Vanderbilt University Medical Center, Nashville, TN, USA; 2 Dermatology Service, Department of Veterans Affairs, Tennessee Valley Healthcare System, Nashville, TN, USA; 3 Department of Pharmacology, Vanderbilt University, Nashville, TN, USA

**Keywords:** Enzymology, lipolysis, fatty acid metabolism, skin, ceramide, triacylglycerol, acyltransferase, skin barrier, epidermis, LC/MS

## Abstract

Loss-of-function mutations in patatin-like phospholipase domain-containing protein 1 (PNPLA1) cause autosomal recessive congenital ichthyosis, and altered PNPLA1 activity is implicated in the pathogenesis of atopic dermatitis and other common skin diseases. To examine the hypothesis that PNPLA1 catalyzes the synthesis of acylceramides and acyl acids, we expressed and partially purified a soluble, truncated form of PNPLA1 in *Escherichia coli*, (PNPLA1_trun_) along with the related protein PNPLA2 (ATGL, adipose triglyceride lipase) and coactivator CGI-58. Liposomal substrates were incubated with recombinant enzymes for 0.5–24 h and products analyzed by HPLC-UV and LC-MS. Using trilinolein or dilinolein substrates, PNPLA1_trun_, like ATGL_trun_, catalyzed lipolysis and acyltransferase reactions with 2–30% conversion into linoleic acid, monolinolein, and trilinolein. CGI-58 enhanced ATGL-catalyzed lipolysis as previously reported, but transacylase activity was not enhanced with ATGL or PNPLA1. In matching the proposed activity in vivo, PNPLA1 catalyzed acyl transfer from trilinolein and dilinolein donors to omega-hydroxy ceramide, omega-hydroxy glucosylceramide, and omega-hydroxy acid acceptors to form acylceramide, glucosyl-acylceramide, and acyl acid, respectively, albeit with only ∼0.05% conversion of the substrates. Notably, in experiments comparing dilinolein vs. diolein acyl donors, PNPLA1 transferred linoleate with 3:1 selectivity over oleate into acylceramide. These results support the role for PNPLA1 in the synthesis of acylceramides and acyl acids in epidermis and suggest that the enrichment of these lipids with linoleic acid could result from the substrate selectivity of PNPLA1.

## Graphical abstract

**Figure:**
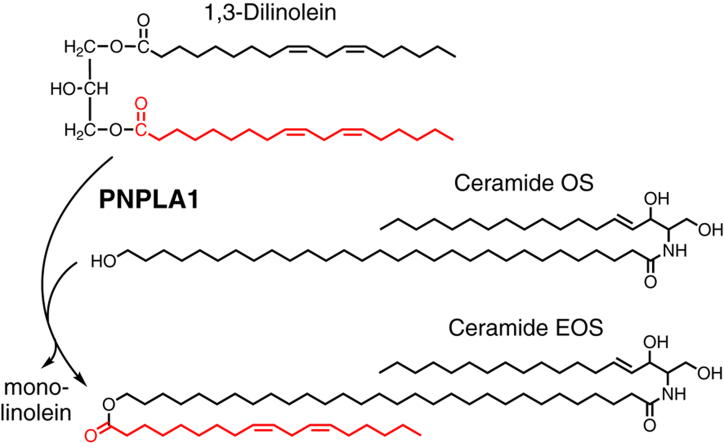

Patatin-like phospholipase domain-containing protein 1 (PNPLA1) is required for skin barrier formation via its proposed role in the synthesis of the ceramide esterified omega-hydroxy acylsphingosine (Cer-EOS) and related acylceramides and acyl acids unique to epidermis (1, 2, 3, 4). In humans, biallelic loss-of-function mutations in PNPLA1 cause autosomal recessive congenital ichthyosis (5, 6), a rare genetic disease with lifelong barrier dysfunction and generalized scaly and inflamed skin. In mice, PNPLA1 deletion results in severe barrier dysfunction and death from dehydration within hours after birth (3). Mutations in PNPLA1 or alpha-beta-hydrolase domain containing five (ABHD5) are responsible for congenital ichthyosis in golden retrievers, in which a common insertion-deletion mutation in PNPLA1 appears to have been spread by in-breeding (7, 8, 9). Several common, acquired human skin diseases also have reductions in acylceramides (10, 11, 12), which might result from altered PNPLA1 expression and/or activity.

PNPLA1 is proposed to transfer fatty acyl groups from triacylglycerol (TAG) to omega-hydroxy ceramides to form acylceramides in a reaction that is promoted by the coactivator comparative gene identification-58 (CGI-58, encoded by the gene *ABHD5*) (2, 13, 14, 15). This hypothesis is consistent with the reduction in epidermal acylceramides and accumulation of the omega-hydroxy ceramide precursors resulting from deletion or loss-of-function mutations in PNPLA1 or ABHD5 (14). Co-expression of PNPLA1, CGI-58, and three other members of the acylceramide synthesis pathway resulted in Cer-EOS synthesis in HEK-293T cells, providing further support for the role of PNPLA1 in Cer-EO synthesis (2). Also, PNPLA1 expressed in a cell-free wheat germ system catalyzed the synthesis of Cer-EOS in subnanogram quantity using the TAG trilinolein but not linoleoyl CoA as an acyl donor (2). Beyond this, little is established about the enzymatic properties of PNPLA1.

PNPLA1 is part of a large family of proteins sharing a common, homologous phospholipase domain. A well-known member of this family is PNPLA2, more commonly known as adipose triglyceride lipase (ATGL) (16). ATGL is activated by CGI-58 (17, 18, 19) and is important in TAG metabolism in adipocytes and other tissues (16). Given significant sequence homology between ATGL and PNPLA1, some similarity of enzymatic activity might be expected. Recently, C-terminal truncation of the ATGL gene was found to facilitate the expression and activity of recombinant protein via increased solubility and/or stability of the protein product (20). In the current report, we used a similar strategy to express recombinant PNPLA1 in order to study its enzymatic activity.

## Materials and methods

### Chemicals and solvents

Dioleoyl-phosphatidylcholine (850375) and dioleoyl-phosphatidylserine (840034) were purchased from Avanti. Monolinolein (M-254), monoolein (M-239), 1,2-dilinolein (D-251), 1, 2-diolein (D-236), trilinolein (T250), and triolein (T-235) were purchased from NuChek Prep. Methyl 30-hydroxy triacontanoic acid (ω-OH-C30:0) was purchased from Nacalai USA (NS490202). Ceramide omega-hydroxy acylsphingosine (Cer-OS) and Cer-EOS were prepared by the Vanderbilt Chemical Synthesis Core. Additionally, Cer-EOS was obtained as a gift from Evonik (Essen, Germany), and Cer-OS (item no. 24394) was from Cayman Chemical (Ann Arbor, MI). HPLC solvents were purchased from Fisher (Waltham, MA). The identity and purity of each sample of Cer-OS and Cer-EOS were confirmed by LC/MS.

### Recombinant protein expression and purification

The complementary DNA (cDNA) construct used for expression of the C-terminal truncated mouse ATGL (ATGL_trun_) matched the 288 amino acid truncated sequence described by Kulminskaya *et al.* (20). The C-terminal truncated human PNPLA1 (PNPLA1_trun_) construct was an equivalent 292 amino acid truncated sequence of human PNPLA1. Both cDNAs (synthesized by Biomatik) were transferred into pET44a and pMAL vectors for expression in *Escherichia coli*. In trial experiments, each gave similar expression results and the pMAL construct was used for routine preparation of the enzymes. Enzymatic cleavage of the maltose-binding protein fusion protein (encoded by the pMAL sequence) did not impact ATGL_trun_ or PNPLA1_trun_ activity (data not shown); therefore, subsequent experiments used the recombinant proteins without removing the maltose-binding protein. Human CGI-58 was cloned from human keratinocyte cDNA and expressed in the pET17b vector with a 5′ 6xHis tag. Constructs were transfected into ArticExpress (DE3) competent cells (Agilent Technologies 230192) according to the manufacturer’s protocol and plated onto LB Agar Miller containing 2% glucose, 100 mg/ml ampicillin, and 20 mg/ml gentamycin. After overnight incubation at 37°C, single colonies were inoculated into 50 ml tubes containing 10 ml LB medium, 2% glucose, 100 mg/ml ampicillin, and 20 mg/ml gentamycin (or the same proportions scaled up to 500 ml) and incubated for 8 h at 37°C with orbital shaking at 250 rpm. 1.75 ml of each 10 ml culture was then transferred into a 500 ml flask containing 75 ml LB, 2% glucose, 100 mg/ml ampicillin, and 20 mg/ml gentamycin and incubated with orbital shaking at 250 rpm at 37°C overnight. Each 50 ml culture was then added to a 2.8 l Fernbach flask containing 915 ml LB, 25 ml 20% glucose, 10 ml 1M MgCl_2_ and incubated at 20°C incubator with orbital shaking at 250 rpm until the absorption at 600 nm reached 0.5. The flask was then cooled at 4°C for 1 h, isopropyl β-d-1-thiogalactopyranoside was added to a final concentration of 0.1–0.5 mM, and incubation continued at 10°C with orbital shaking at 150 rpm for 20–24 h. The final cultures were centrifuged in 400 ml centrifuge bottles at 4,650 g at 4°C for 1 h and the supernatant discarded. The pellets were resuspended in 50 mM Tris pH 7.9, combined into a 50 ml tube, and centrifuged at 4,600 g for 30 min at 4°C. The supernatant was discarded, the pellet resuspended in 25 ml 1 × TSE buffer containing 1 mg/ml egg yolk lysozyme, and placed on an orbital mixer at 4°C for 30 min. After centrifugation again at 4,600 g for 30 min at 4°C, the supernatant was discarded, and the pellet frozen at −80°C overnight.

### Nickel affinity column purification

Frozen pellets of bacteria expressing PNPLA1, ATGL, or CGI-58 were resuspended on ice in 20 ml lysis buffer (100 mM potassium phosphate buffer pH 7.5, 100 mM KCl, 10% glycerol (v/v), 0.1% Nonidet P40 (v/v), 30 mM imidazole, 1 mM ATP, 1 mM TCEP, 2 ml benzonase, 10 mM MgCl_2_, and Roche Protease inhibitor mix, EDTA free). The pellet was disrupted using a Fisher Scientific Model 100 Sonic Dismembrator on power setting 9 with sonication on ice for 6 × 20 s intervals with 20 s between each sonication cycle. After passing through 10 strokes of a glass homogenizer on ice, the sample was placed on an orbital mixer at 4°C for 1 h and then centrifuged at 16,000 g for 30 min at 4°C. The supernatant was loaded onto a 1.2 ml NiNTA (Qiagen) resin column in a 10 ml column support at 4°C and run by gravity flow. The column was washed as follows: *i*) 6 ml of lysis buffer, *ii*) 8.5 ml of wash buffer (100 mM K_3_PO^4^ pH7.5, 500 mM KCl, 10% glycerol, 30 mM imidazole, 1 mM TCEP, 0.003% Nonidet P40, 10 mM ATP, and 10 mM MgCl2), *iii*) a final wash using 12 ml equilibration buffer (100 mM K_3_PO_4_ pH 7.5, 100 mM KCl, 1 mM TCEP, 10% glycerol), *iv*) elution using four aliquots collected separately of 900 ml elution buffer (100 mM K_3_PO_4_ pH 7.5, 100 mM KCl, 1 mM TCEP, 0.003% Nonidet P40, 500 mM imidazole). The second elution fraction (“E2”), which typically contained the highest concentration of protein, was used for experiments and the other fractions discarded. The proteins were then dialyzed overnight into 50 mM K_3_PO_4_ pH 7.5, 100 mM NaCl, 0.003% Nonidet P40, 10% glycerol and stored at −80°C.

### Synthesis of 30-linoleoyl-oxy-triacontanoic acid (acyl acid)

Methyl 30-hydroxy-triacontanoate (2 mg, Nacalai USA NS490202) was hydrolyzed to the free acid by dissolving in 200 μl CHCl_3_ in an 8 ml glass Schwartz vial (warming required), followed by addition of 800 μl 1M KOH in 95% methanol and heating at 65°C for 2 h with vigorous mixing at 15-min intervals. The free acid was extracted with CHCl_3_ after addition of water (2 ml) and 1M HCl titrated to pH 4.0. The CHCl_3_ phase was collected, washed with water, and the sample taken to dryness under nitrogen in a 1 ml Reacti-vial. Pyridine (40 μl) and linoleoyl anhydride (6 μl, TCI L0145) were added, and the sample was incubated under argon in a 55°C water bath overnight. The reaction progress was monitored by TLC (HL TLC plate, Analtech 43931-2) using a mobile phase on hexane/ethyl acetate/glacial acetic acid (80:20:1) in comparison to linoleic acid (LA) and linoleoyl anhydride standards and visualized using CuSO_4_ spray and heating to 180°C for 15 min. The 30-linoleoyl-oxy-triacontanoic acid (ω-OH-C30:0-linoleate) was purified by HPLC using a Kinetex C8 5 μm column (4.6 × 250 mm) with a mobile phase of methanol/hexane/acetic acid 95:5:0.02 and a flow rate of 1 ml/min. The expected molecular mass of the product was confirmed by LC-MS ([M-H]^−^, found 729.6773, calc. 729.6766).

### Preparation of Glucosyl-Cer-OS as substrate for PNPLA1

Glucosyl-Cer-EOS (Glc-Cer-EOS) was isolated from pig epidermis as follows. 20 × 20 cm sections of pig skin were collected within 1 h of animals being euthanized after unrelated experiments (the skin would have otherwise been discarded) in the Department of Surgery and the tissue frozen at −80°C. Epidermis was separated by incubation of the skin at 65°C for 1 min, and lipids extracted from epidermis with CHCl_3_/methanol and purified by straight phase-HPLC (SP-HPLC) using a TLC Advantage silica column (5 μm, 4.6 × 250 mm) running at 1 ml/min and using solvents (A) hexane/isopropanol/acetic acid 95:5:0.1 and (B) hexane/isopropanol/acetic acid 75:25:0.1 on a gradient of 100% A to 100% B over 30 min with online UV detection at 205 nm. To prepare Glucosyl-Cer-OS (Glc-Cer-OS), the esterified fatty acid moiety in Glc-Cer-EOS (mainly linoleate), was removed by alkaline hydrolysis as follows. Glc-Cer-EOS (1.5 mg) was dried in a 50 ml glass tube and resuspended in 500 μl CHCl_3_. 4.5 ml of 1M KOH in 95% methanol was added and the sample stirred at 50°C for 2 h. The ceramide was initially in solution but precipitated as the fatty acid was released. The mixture containing the Glc-Cer-OS product was neutralized and slightly acidified by the addition of 1 ml 1M KH_2_PO_4_ and sufficient 1M HCl to achieve pH 4 and then extracted with CHCl_3_/methanol/water in the Bligh & Dyer proportions (21). The lower CHCl_3_ phase was collected, and the upper phase re-extracted using theoretical lower phase (prepared by mixing and partitioning the pure solvents). Both lower phases were combined and taken to dryness. The Glc-Cer-OS was purified on SP-HPLC on a TLC silica column (5 μm, 4.6 × 250 mm) using isocratic elution with hexane/isopropanol/acetic acid 75:25:0.1 and a flow rate at 1 ml/min. Although Glc-Cer-OS, having lost the linoleate ester, exhibits relatively weak UV absorbance at 205 nm compared to the starting Glc-Cer-EOS analog, the large amounts injected gave a readily detectable signal and the expected mass spectrum of the isolated product was confirmed by LC-MS.

### Micelle preparation

Micelles containing trilinolein or dilinolein were prepared by drying the premixed lipids (879 μg 1,3-dilinolein or trilinolein, 56.9 μg dioleoyl phosphatidylcholine, and 19.6 μg palmitoleoyl phosphatidylserine) onto the sides of a 5 ml glass vial under a nitrogen stream. Two milliliters of 100 mM HK_2_PO_4_ buffer (pH 7.4) was added to the tube, and the resulting oil droplets were dispersed into micelles by ultrasonication using a probe sonicator (Sonic Dismembrator Model 100, Fisher Scientific, Waltham, MA) at 20% power for 1 min on ice, in 5 s intervals with equal rest intervals between each sonication, resulting in a stable, slightly cloudy emulsion.

### Liposome preparation

Phosphatidylcholine liposomes containing TAG or diacylglycerol and Cer-OS were prepared by drying the premixed lipids (3 mg dioleoyl phosphatidylcholine, 830 μg palmitoleoyl phosphatidylserine, 150 μg Cer-OS, and 150 μg of trilinolein, dilinolein, or dilolein) onto the sides of a 1.5 ml eppendorf tube under a nitrogen stream followed by vacuum desiccation overnight at RT. The dried sample was resuspended in 1 ml degassed Dulbecco’s PBS pH 7.4 without CaCl_2._ The tube was flushed with argon and warmed in 37°C with vortex mixing at 5 min intervals. The tube was then placed in a water bath sonicator (Branson 3200) and sonicated every other minute for 10 min. This resulted in a stable, milky suspension of crude liposomes. An Avanti Mini Extruder was assembled with 10 mm filter supports (610014) and a polycarbonate membrane 0.1 μm, 19 mm (610005). The block was warmed to 37°C. The crude liposome preparation was drawn into the 1 ml glass syringe provided with the extruder kit and pushed through the membrane filter 20 times, resulting in a transparent solution. Liposomes prepared with ω-OH-C30:0 would not pass through the extruder, even at 50°C. In this case, the crude liposomes were used directly for reactions.

### Transacylase and lipolysis reactions

For each reaction, recombinant enzymes (or recombinant enzymes boiled for 10 min on a plate-heater) were combined with micelles or liposomes in 1.5 ml eppendorf tubes so that the final reaction volume resulted in a 2-fold dilution of the micelles/liposomes. The tubes were incubated at 37°C on an orbital shaking heater plate for 2 h or for the indicated time period. The reactions were terminated by the addition of methanol. Lipid products were extracted by the Bligh and Dyer method (methanol:CHCl_3_:water, final ratio 2:2:1.8 by volume (21)). The lower organic phase was collected with a 1 ml Hamilton syringe. The upper phase was re-extracted with an equivalent volume of “theoretical” Bligh and Dyer lower phase (prepared by mixing solvents in the final Bligh and Dyer proportions) and the lower phase again collected. The combined organic phases were dried and resuspended in solvents as indicated for HPLC and/or LC-MS.

### HPLC-UV analyses

Aliquots of lipids were analyzed by reverse phase-HPLC using a Waters Symmetry C18 column (4.6 × 250 mm) or a Kinetex C8 column (4.6 × 250 mm), a running solvent of methanol/hexane/acetic acid (90/10/0.02 or 95/5/0.02 by volume), at a flow rate of 1 ml/min, with on-line UV detection (Agilent 1100 series diode array detector). To facilitate identification of the spectrum of naturally occurring species in pig skin Glc-Cer-EOS and Glc-Cer-OS, these lipids were analyzed by SP-HPLC using a Thomson Advantage 5 μm silica column (4.6 × 250 mm) using a solvent of hexane/isopropanol/acetic acid (90/10/0.02, by volume) run at 1 ml/min.

### LC-MS analyses

LC-MS profiles were obtained by reverse-phase LC-MS with electrospray ionization using a Thermo Scientific Q Exactive Focus LC-MS/MS (Thermo Fisher Scientific, Waltham, MA). A Kinetex C8 column (4.6 × 250 mm) was used for the HPLC with a running solvent of methanol/hexane/acetic acid (90/10/0.02 or 95/5/0.02 by volume) at a flow rate of 1 ml/min. The negative ion mode was used to analyze fatty acids, while the positive ion mode was used to analyze acylglycerols and ceramides. The electrospray voltage was set at 4.0 kV: vaporizer temperature at 300°C; sheath and auxiliary gas pressure at 50 and 5 ψ, respectively, and capillary temperature at 300°C. Glc-Cer-EOS and Glc-Cer-OS were analyzed by straight phase LC-MS with an Alliance 2690 HPLC system (Waters Corporation, Milford, MA) coupled to a TSQ Vantage mass spectrometer (Thermo Fisher Scientific, Waltham, MA) with a Grace Altima SI 5 μm column (2.1 × 100 mm) and a running solvent of hexane/isopropanol/acetic acid (75/25/0.1, by volume) run at 0.3 ml/min and a Thomson Advantage 150A 5-μm silica column (250 × 4.6 mm) with a solvent of hexane/isopropanol (100:1, v/v) and a flow rate of 1 ml/min. The APCI vaporizer temperature was set to 350°C, sheath and auxiliary gas pressure at 50 and 5 ψ, respectively, and capillary temperature at 300°C. Absolute quantification (expressed as pmol of product per reaction) was determined from the LC/MS chromatogram peak area in comparison to the corresponding authentic standard.

### Statistical analysis

Statistical tests were performed with Prism 9 (GraphPad, San Diego, CA). Normality of sample distribution was determined by the Shapiro-Wilk test. Unpaired *t*-tests with Welch’s correction (not assuming equal standard deviations) were used to test differences between groups with normally distributed data.

## Results

### Production of recombinant ATGL, CGI-58, and PNPLA1 proteins

**Fig. 1:**
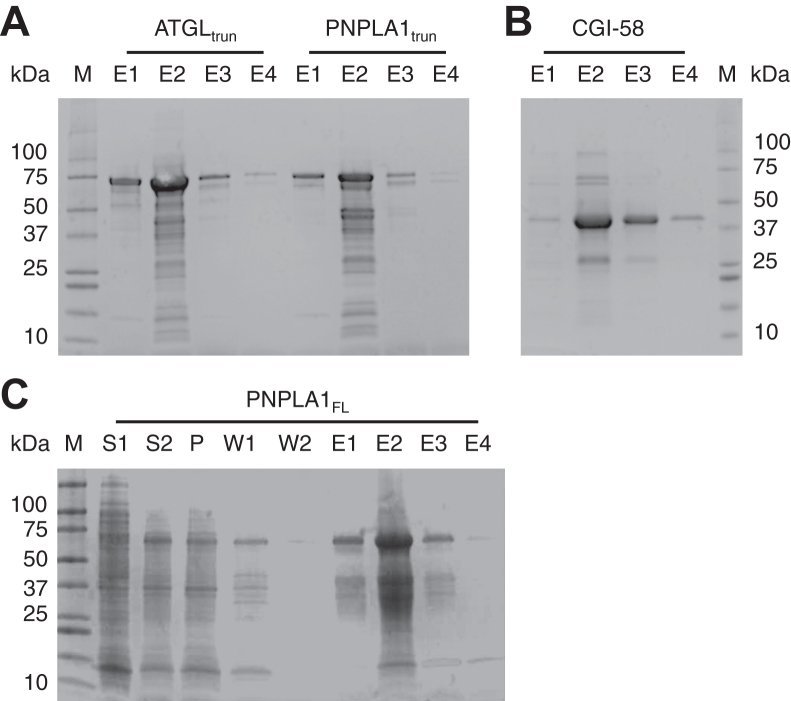
SDS-PAGE analysis of ATGL, PNPLA1, and CGI-58. Recombinant His-tagged ATGL_trun_ and PNPLA1_trun_ (A), CGI-58 (B), and PNPLA1_FL_ (C) were expressed in *Escherichia coli* and purified by immobilized nickel affinity chromatography. Proteins eluting from the column in wash buffer (W1, W2) or elution buffer (E1, E2, etc) or in the supernatant (S1, S2) or pellet (P) from the lysed bacterial cells were separated by SDS-PAGE and stained with Coomassie blue. The E2 fraction of each purification was used for subsequent experiments. Expected molecular weight (including the mannose-binding protein fusion peptide where present): ATGL_trun_, 73 kDa; PNPLA1_trun_, 75 kDa; CGI-58, 39 kDa; PNPLA1_FL_, 58 kDa. M, molecular weight marker. ATGL, adipose triglyceride lipase; CGI, comparative gene identification.

Recombinant murine ATGL with the C-terminus truncated to 288 amino acids as described (20) (ATGL_trun_) and an equivalent C-terminal truncated 292 amino acid construct of human PLPLA1 (PNPLA1_trun_) were expressed in *E. coli* and recovered from the lysed bacteria with the addition of low concentrations of the nonionic, nondenaturing detergent Nonidet P-40. Full-length human PNPLA1 (PNPLA1_FL_) could be solubilized with the further addition of the ionic detergent Fos Chol 12. Recombinant human CGI-58 was recovered in an aqueous buffer containing no detergents. The recombinant proteins were partially purified by nickel affinity chromatography with major protein bands appearing at the expected molecular weight (including the maltose binding protein fusion, where present) by SDS-PAGE (Fig. 1).

### Characterization of transacylase and lipase activities of ATGL_trun_ and PNPLA1_trun_

**Fig. 2:**
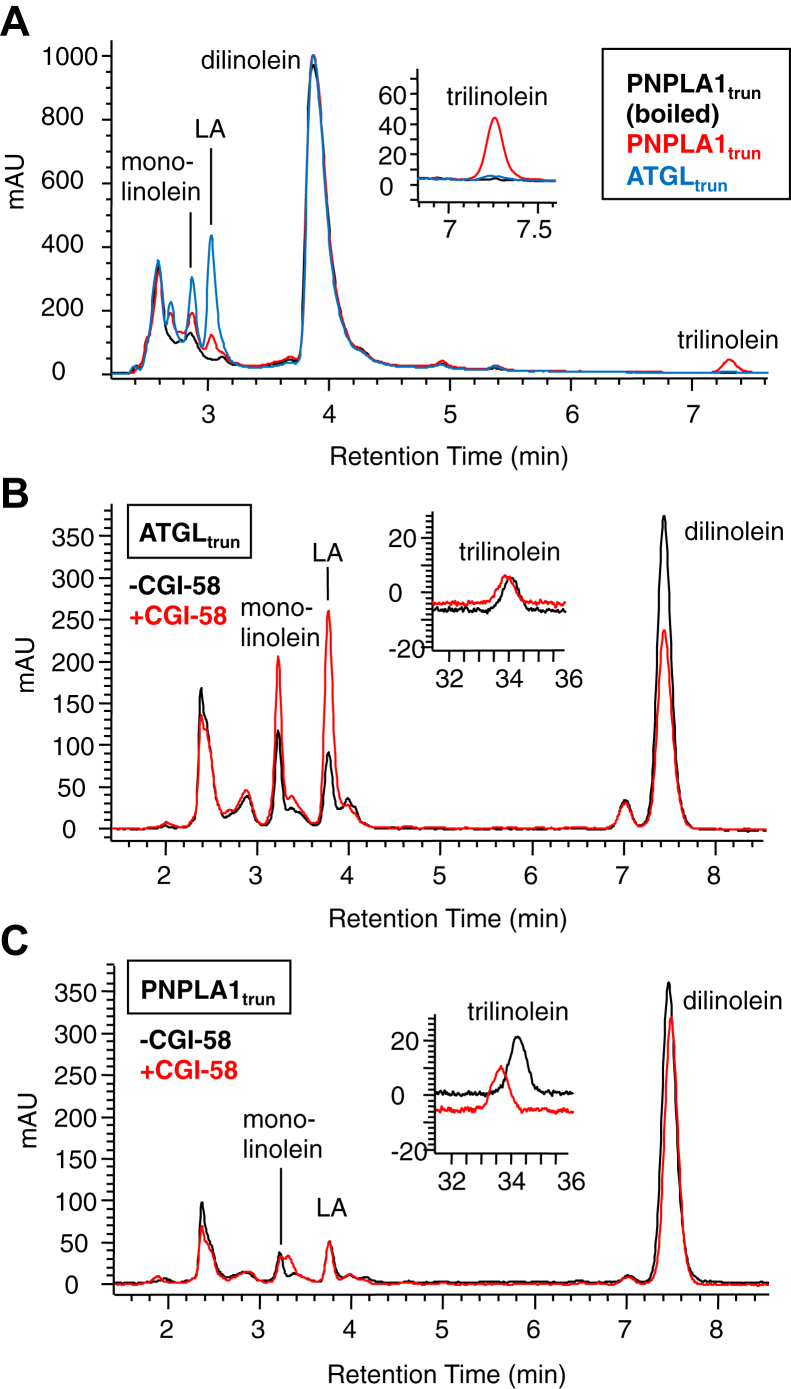
Transformations of dilinolein by ATGL_trun_, PNPLA1_trun_, and CGI-58 as analyzed by HPLC-UV. The indicated proteins were incubated with CGI-58 and dilinolein liposomes (A) or with dilinolein micelles ± CGI-58 (B, C) for 2 h at 37°C, then the reaction products were extracted into CHCl_3_, dried and resuspended in methanol/hexane/acetic acid 90:10:0.02, and analyzed by isocratic RP-HPLC with online UV detection (205 nm absorption shown) using a Kinetex 4.6 × 250 mm C8 column (A) or a Waters Symmetry 4.6 × 250 mm C18 column (B, C). Monolinolein (ML), LA, dilinolein, and trilinolein were identified by retention time in comparison to authentic standards. CGI, comparative gene identification; LA, linoleic acid; RP-HPLC, reversed phase-HPLC.

Incubation of ATGL_trun_ or PNPLA1_trun_ and CGI-58 with liposomal dilinolein for 2 h led to the formation of lipolysis and transacylation products detectable by HPLC-UV (Fig. 2A). Monolinolein and LA were the major lipolysis products formed in reactions with ATGL_trun_, while the transacylation product trilinolein (Fig. 2, insert) was a major product formed in reactions with PNPLA1_trun_. Product formation was prevented by boiling of the proteins prior to incubation with the liposomes. Similar reactions were observed when the enzymes were incubated with dilinolein micelles rather than liposomes (Fig. 2B, C). CGI-58 enhanced the formation of monolinolein and LA by ATGL_trun_ but did not affect the formation of trilinolein (Fig. 2B), as previously reported (20). The addition of CGI-58 to reactions with PNPLA1_trun_ did not appreciably affect any of the products (Fig. 2C). The identity of the monolinolein, LA, and trilinolein reaction products were confirmed by LC-MS (supplemental Fig. S1). In contrast to the truncated protein, the full-length recombinant human PNPLA1_FL_ did not appear to be active by HPLC-UV (not shown) or LC-MS (supplemental Fig. S2) analysis vs. boiled control enzyme, as previously reported for full-length recombinant murine ATGL (20).

### Synthesis of Cer-EOS by PNPLA1_trun_

**Fig. 3:**
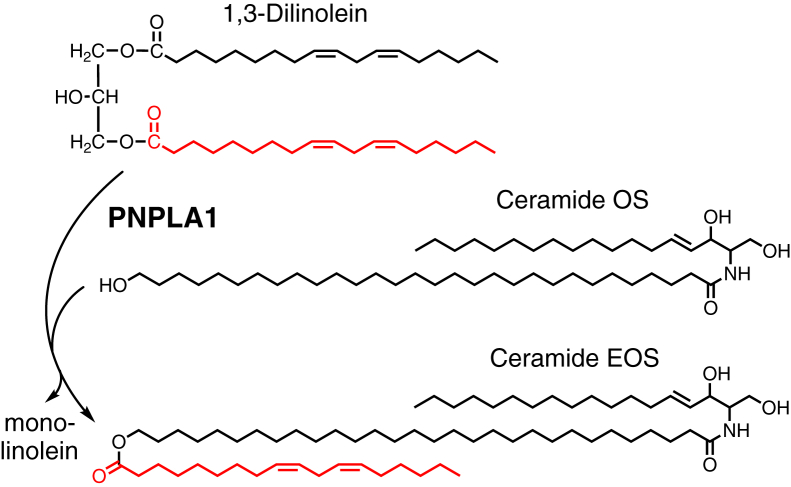
Transacylase activity in acylceramide synthesis. Reaction scheme for the transfer of linoleate from dilinolein to ceramide OS to form the acylceramide Cer-EOS and monolinolein (ML). Cer-EOS, ceramide esterified omega-hydroxy acylsphingosine.

**Fig. 4:**
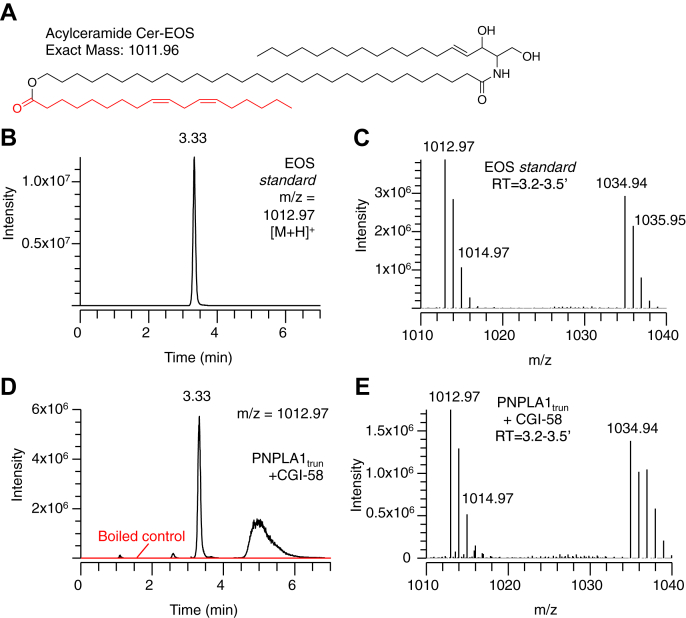
Synthesis of the acylceramide Cer-EOS by PNPLA1_trun_. A: Structure and exact mass of Cer-EOS standard. B: Reversed phase LC/MS Chromatogram of Cer-EOS standard (m/z = 1012.97). C: Mass spectrum of the major peak from 3.2–2.5 min. D, E: LC/MS chromatogram (D) and mass spectrum (E) of Cer-EOS synthesized in 2 h at 37°C by PNPLA1_trun_ and CGI-58 from liposomes containing dilinolein and Cer-OS in comparison to boiled control proteins. Use of trilinolein in place of dilinolein gave similar product formation (not shown). See Materials and Methods for LC/MS conditions. Cer-EOS, ceramide esterified omega-hydroxy acylsphingosine; Cer-OS, ceramide omega-hydroxy acylsphingosine; CGI, comparative gene identification.

Transfer of acyl groups from acylglycerols to Cer-OS results in the synthesis of Cer-EOS (Fig. 3). The synthetic EOS standard (Fig. 4A), also an abundant natural species in epidermis, could be detected by LC/MS in positive ion mode as ions with masses corresponding to [M+H]^+^ and [M+Na]^+^ (Fig. 4B, C), as reported (22). Lipids containing ultra-long saturated acyl chains (Cer-OS and ω-OH-C30:0) formed large crystalline precipitates during micelle preparation but were successfully incorporated into liposomes. Incubation of PNPLA1_trun_ and CGI-58 with liposomes containing Cer-OS and either dilinolein or trilinolein led to the formation of a Cer-EOS product matching the retention time and mass spectrum of the Cer-EOS standard (Fig. 4D, E). Similar to the reactions with dilinolein liposomes without Cer-OS, acylglycerol and free fatty acid products were also detected (not shown). As with trilinolein synthesis, Cer-EOS formation by PNPLA1_trun_ was not significantly affected by the addition of CGI-58 (supplemental Fig. S3). Traces quantities of Cer-EOS product were also detected in reactions with PNPLA1_FL_, although the quantities were substantially lower than those with PNPLA1_trun_ (supplemental Fig. S4).

### Synthesis of linoleoyl acyl acid by PNPLA1_trun_

**Fig. 5:**
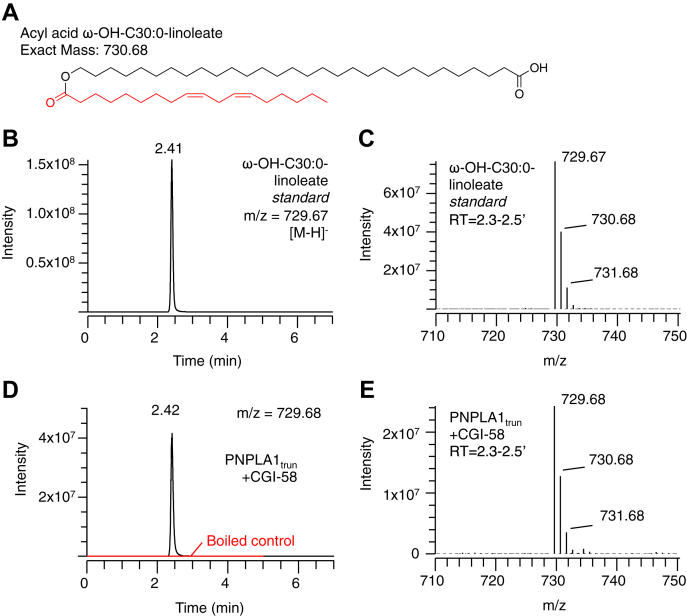
Synthesis of the acyl acid ω-OH-C30:0-linoleate by PNPLA1_trun_. A: Structure and exact mass of the acyl acid ω-OH-C30:0-linoleate. B, C: Reversed phase LC/MS chromatogram (B) and mass spectrum (C) of the ω-OH-C30:0-linoleate standard. D, E: Reversed phase LC/MS chromatogram (D) and mass spectrum (E) of ω-OH-C30:0-linoleate synthesized by PNPLA1_trun_ and CGI-58 or boiled control proteins (2 h, 37°C) from liposomes containing dilinolein and ω-OH-C30:0. See Materials and Methods for LC/MS conditions. CGI, comparative gene identification.

Acyl transfer from acylglycerols to ω-OH fatty acids results in the formation of acyl acids, which are major lipid constituents in epidermis related to acylceramides and protein-bound lipid (23). In order to study whether PNPLA1 catalyzes acyl acid synthesis, we synthesized an acyl acid standard (ω-OH-C30:0-linoleate), confirming its identity by LC-MS (Fig. 5A–C). Incubation of PNPLA1_trun_ with liposomal ω-OH-C30:0 and dilinolein substrates resulted in the formation of a ω-OH-C30:0-linoleate product matching the retention time and mass spectrum of the authentic standard (Fig. 5D, E). In control reactions, there was no product formation using boiled enzyme.

### Time course of PNPLA1_trun_-catalyzed acyltransferase reactions

**Fig. 6:**
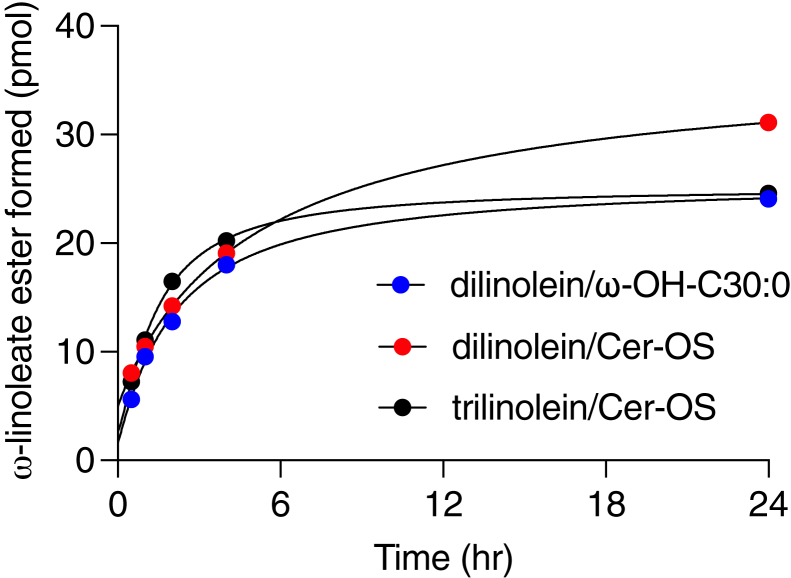
Time course of linoleoyl transfer by PNPLA1_trun_ and CGI-58. PNPLA1_trun_ and CGI-58 were incubated at 37°C with liposomes containing the indicated lipids. After the indicated time period, the reaction was terminated by the addition of methanol, and the reaction products (ω-OH-C30:0-linoleate acyl acid for the dilinolein/ω-OH-C30:0 reaction and Cer-EOS for the dilinolein/Cer-OS and trilinolein/Cer-OS reactions) were extracted and quantified by reversed phase LC/MS vs. authentic standards. See Materials and Methods for LC/MS conditions and methods used for quantification. Cer-EOS, ceramide esterified omega-hydroxy acylsphingosine; Cer-OS, ceramide omega-hydroxy acylsphingosine; CGI, comparative gene identification.

To compare reaction rates with different substrates, the same molar concentration of the acyl donor (dilinolein or trilinolein) and omega-hydroxy acyl acceptor (ω-OH-C30:0 or Cer-OS) was used in each reaction (details in Materials and Methods). With each substrate combination, the corresponding linoleoyl ester product accumulated progressively for 4 h, after which the rate of product accumulation decreased (Fig. 6). The moles of product formed were similar whether trilinolein or dilinolein was used as the acyl donor and whether ω-OH-C30:0 or Cer-OS was used as the acyl acceptor. The quantities of products formed by PNPLA1_trun_ in acylceramide and acyl acid synthesis amounted to only ∼0.05% molar conversion of substrates, compared to more than 5% conversion in the acyltransferase of dilinolein into trilinolein.

### Comparison of PNPLA1_trun_ versus ATGL_trun_ in the incorporation of linoleate versus oleate into Cer-EOS

**Fig. 7:**
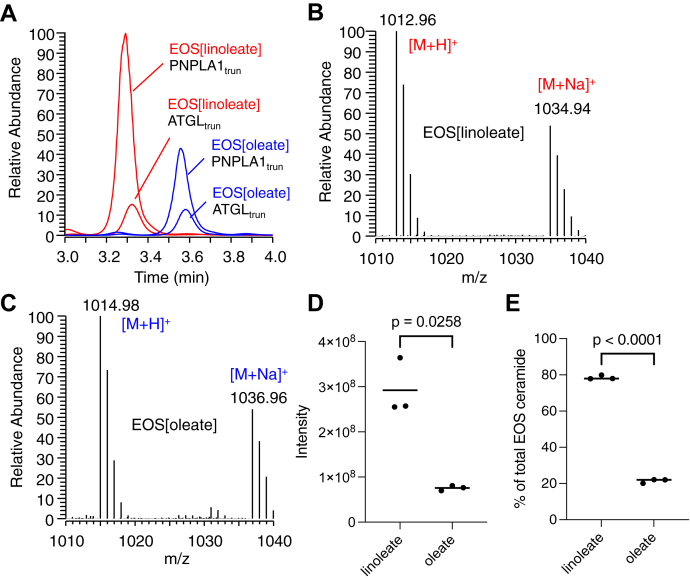
Greater activity and acyl group selectivity of PNPLA1_trun_ versus ATGL_trun_ in the synthesis of Cer-EOS. A–D: Recombinant CGI-58 and either PNPLA1_trun_ or ATGL_trun_ were incubated with liposomes containing Cer-OS and either dilinolein (red) or diolein (blue) for 2 h at 37°C, and reaction products were extracted and analyzed by LC/MS. A: Overlay of reversed phase LC/MS chromatograms (m/z = 1010–1040) of products from reactions with the indicated enzymes and substrates. B: Mass spectrum of Cer-EOS (RT = 3.15–3.5 min) formed by PNPLA1_trun_ from dilinolein/Cer-OS liposomes. C: Mass spectrum of Cer-EOS (RT = 3.4–3.7 min) formed by PNPLA1_trun_ from diolein/Cer-OS liposomes. D: Quantification of Cer-EOS[linoleate] synthesized by PNPLA1_trun_ from dilinolein/Cer-OS liposomes vs. Cer-EOS[oleate] synthesized from diolein/Cer-OS liposomes. E: Quantification of Cer-EOS[linoleate] and Cer-EOS[oleate] synthesized by PNPLA1_trun_ and CGI-58 (37°C, 2 h) from liposomes containing Cer-OS, dilinolein, and diolein. See Materials and Methods for LC/MS conditions. Cer-EOS, ceramide esterified omega-hydroxy acylsphingosine; Cer-OS, ceramide omega-hydroxy acylsphingosine; CGI, comparative gene identification.

Like PNPLA1, ATGL catalyzed the synthesis of Cer-EOS from Cer-OS and dilinolein or trilinolein (Fig. 7A). In three independent experiments, PNPLA1_trun_ synthesized more Cer-EOS than a matched molar concentration of ATGL_trun_. As expected in these reversed-phase LC/MS analyses, Cer-EOS[linoleate] elutes before Cer-EOS[oleate] (Fig. 7A) with two mass units difference between the two (Fig 7B, C). The amount of Cer-EOS product formed by PNPLA1_trun_ from dilinolein was 3.62-fold greater than that formed from diolein, while no such difference was apparent in the incubations with ATGL_trun_ (Fig. 7A, D). When PNPLA1_trun_ was incubated with liposomes containing both dilinolein and diolein in equal quantity, 3.66-fold more linoleate-containing Cer-EOS was formed than oleate-containing Cer-EOS (Fig. 7E).

### Synthesis of glycosyl EOS

**Fig. 8:**
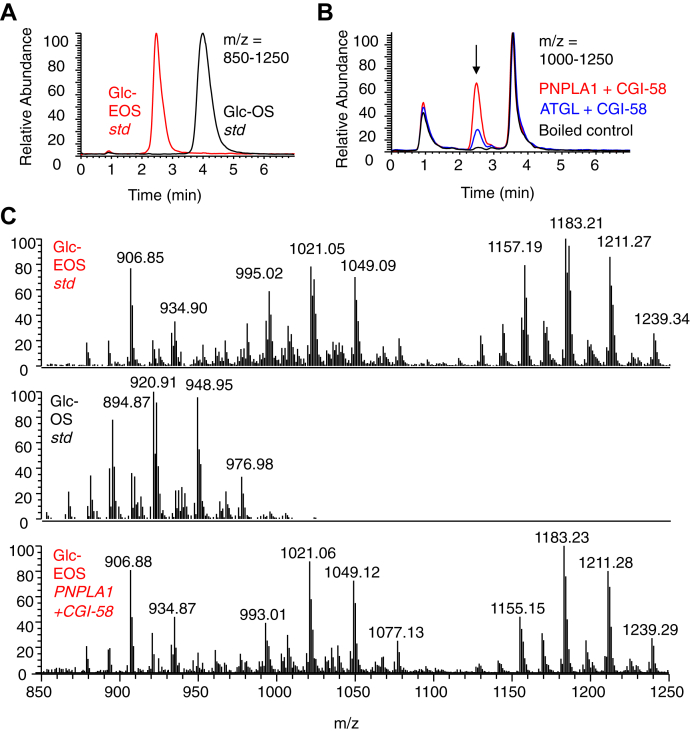
Synthesis of Glucosyl EOS by PNPLA1_trun_ and ATGL_trun_. Glucosyl Cer-EOS (Glc-Cer-EOS) and glucosyl Cer-OS (Glc-Cer-OS) standards were prepared from pig skin as described in Materials and Methods. Recombinant CGI-58 and either PNPLA1_trun_ or ATGL_trun_ were incubated with liposomes containing dilinolein and Glc-Cer-OS for 2 h at 37°C. A: Normal-phase HPLC analyses (silica column, solvent hexane/isopropanol/glacial acetic acid, 90:10:0.02 v/v/v) with an overlay of LC/MS chromatograms of the Glc-Cer-EOS and Glc-Cer-OS standards (m/z = 850–1250). B: Overlay of LC/MS chromatograms (m/z = 1000–1250) of products from the indicated reactions with CGI-58 and either PNPLA1_trun_, ATGL_trun_, or boiled PNPLA1_trun_ control. The retention time of the Glc-Cer-EOS standard is indicated by the arrow. C: Mass spectra of Glc-Cer-EOS standard (RT = 2–3.5 min), Glc-Cer-OS standard (RT = 3.5–5 min), and Glc-Cer-EOS (RT = 2–3.5 min) formed by PNPLA1_trun_ from liposomes containing dilinolein and Glc-Cer-OS. See Materials and Methods for LC/MS conditions. Cer-EOS, ceramide esterified omega-hydroxy acylsphingosine; Cer-OS, ceramide omega-hydroxy acylsphingosine; CGI, comparative gene identification.

Epidermal ceramides, including Cer-EOS, are glycosylated in epidermal keratinocytes and later deglycosylated after secretion into the extracellular space in the superficial epidermis (24). To determine whether PNPLA1 can utilize glucosylceramide substrates, Glc-Cer-OS and Glc-Cer-EOS standards were prepared from pig skin and their identities confirmed by LC-MS (Fig. 8A). When the Glc-Cer-OS substrate was incorporated into liposomes with dilinolein and incubated with CGI-58 and either PNPLA1_trun_ or ATGL_trun_, formation of a family of Glc-Cer-EOS products that matched the retention time (Fig. 8B, Glc-Cer-EOS indicated by ∗) and mass spectrum (Fig. 8C) of the Glc-Cer-EOS standard were detected. The amount of product formed by PNPLA1_trun_ was >3 times greater than that formed by ATGL_trun_ (Fig. 8B).

## Discussion

In this report, we demonstrate that recombinant PNPLA1_trun_ protein catalyzes the synthesis of acylceramide and acyl acid. As previously reported for ATGL (20), C-terminal truncation of the PNPLA1 gene greatly increased the solubility and activity of the expressed protein. Acyltransferase and hydrolase activities of PNPLA1_trun_ with acylglycerol substrates were comparable to ATGL_trun_, and the products were sufficiently abundant to be readily detectable by HPLC-UV (Fig. 2). By contrast, the amounts of acylceramide and acyl acid products formed by PNPLA1_trun_ were ∼2 orders of magnitude smaller and could only be detected by LC-MS (Fig. 4, Fig. 5, Fig. 6, Fig. 7, Fig. 8; supplemental Figs. 3 and 4). The reason for such a difference in these in vitro incubations is not clear, although this does not rule out greater activity in the synthesis of acylceramides and acyl acids in vivo. Lipolytic activity was greater with ATGL_trun_, while transacylase activity, including the synthesis of Cer-EOS, was greater with PNPLA1_trun_.

PNPLA1-inactivating mutations or gene deletion result in nearly complete loss of epidermal acylceramides and acyl acids (1, 2, 3, 4, 6), and therefore ATGL does not appear to have an important role in the synthesis of these lipids in vivo. This could relate in part to the differences in enzyme activity we describe and/or to differences in PNPLA1 and ATGL expression in epidermal keratinocytes. In line with a previous report on the stimulatory effects of CGI-58 on ATGL, we found that CGI-58 greatly enhanced ATGL-catalyzed lipolysis but did not demonstrably affect the transacylase activity forming TAG or acylceramide. CGI-58 gene deletion in vivo results in a severe skin barrier defect, and the available evidence supports its essential role in the synthesis of acylceramides (1). Nonetheless, the mechanism by which CGI-58 promotes transacylase activity in vivo is not known and might involve factors that could not be addressed in this study, such as the appropriate localization of PNPLA1 to lipid droplets (14, 15).

The recombinant PNPLA1_trun_ protein allowed for an initial characterization of substrate specificity in transacylase reactions. In these in vitro experiments, dilinolein and trilinolein were comparable as acyl donors, and the transacylase recipient could be acyl acid, Cer-OS, or Glc-Cer-OS. The presence or absence of large functional groups (sphingoid base or glucosyl-sphingosine) on the carbonyl end of the acyl acceptor had little apparent effect. Whether the glucosylation reaction occurs on OS or EOS is a point our experiments do not address. The results are compatible with glucosylation occurring either before EOS synthesis (Cer-OS → Glc-Cer-OS → Glc-Cer-EOS) or after Cer-EOS synthesis (Cer-OS → Cer-EOS → Glc-Cer-EOS). On the specificity for the fatty acid esterified to the recipient omega-hydroxyl, PNPLA1_trun_ was distinct from ATGL_trun_ in demonstrating a selectivity for the transfer of LA over oleic acid (Fig. 7). This could explain why epidermal acylceramides, but not TAG or phospholipids, are enriched in LA (23). Interestingly, the residual acylceramides present in mice with *Pnpla1* deletion contain mainly oleic acid and saturated fatty acids (3) and therefore might be formed by ATGL or other acyltransferases in the absence of PNPLA1.

## Data availability

No data sets were generated in this study. Raw data is available by request from the corresponding authors.

## Supplemental data

This article contains supplemental data.

## Conflicts of interest

The authors declare that they have no conflicts of interest with the contents of the article.
