# Supplementary material for: Recombinant PNPLA1 catalyzes the synthesis of acylceramides and acyl acids with selective incorporation of linoleic acid
Source: J Lipid Res. 2023 Apr 21;64(6):100379. doi: 10.1016/j.jlr.2023.100379 (PMC10209018; doi:10.1016/j.jlr.2023.100379)
Supplement: Supplemental Figures S1–S4 [file mmc1.pdf]

Supplemental information

Recombinant PNPLA1 catalyzes the synthesis of acylceramides and acyl acids with selective incorporation of linoleic acid

Jason M. Meyer, William E. Boeglin, Alan R. Brash

Figures S1 – S4

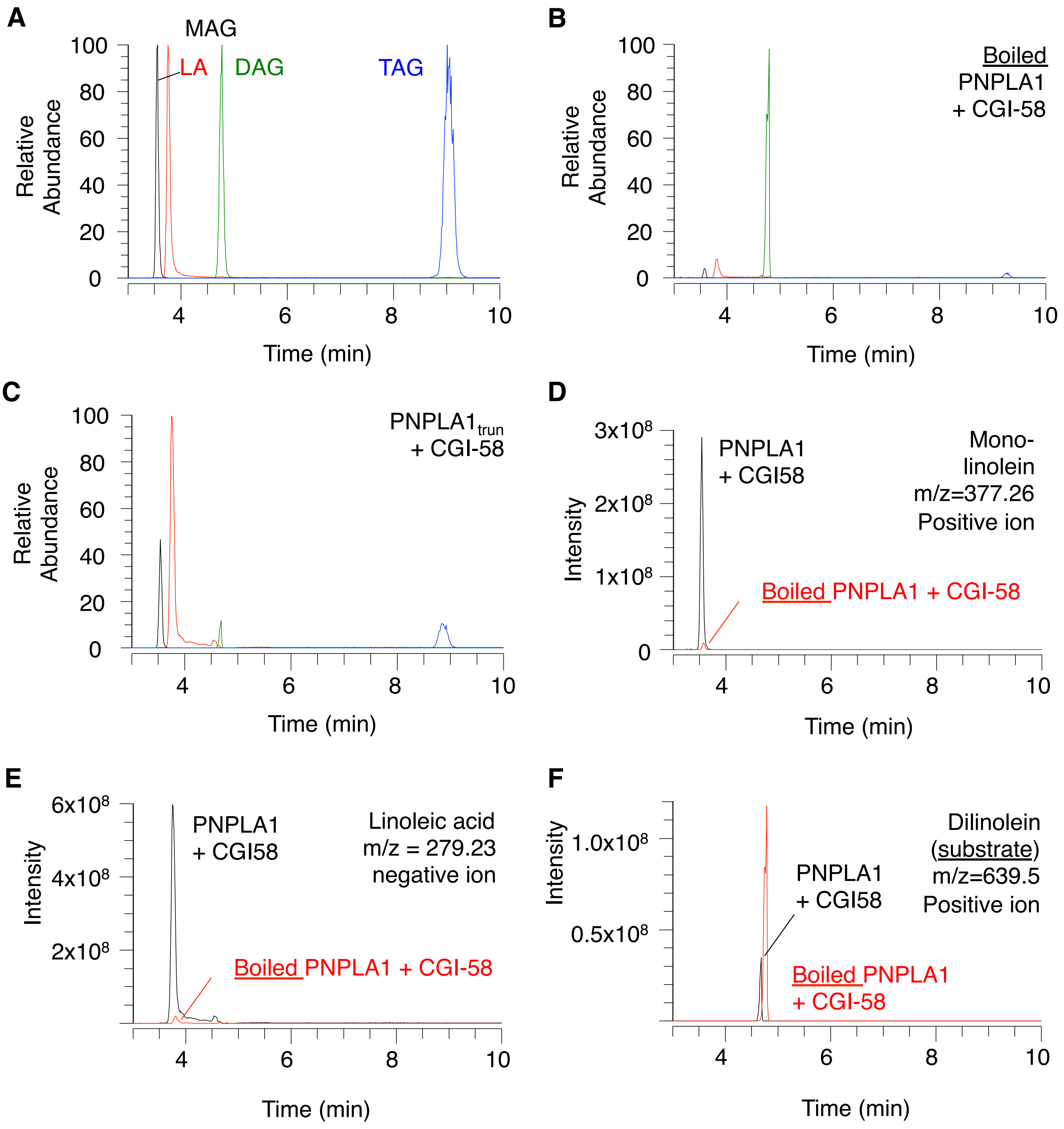

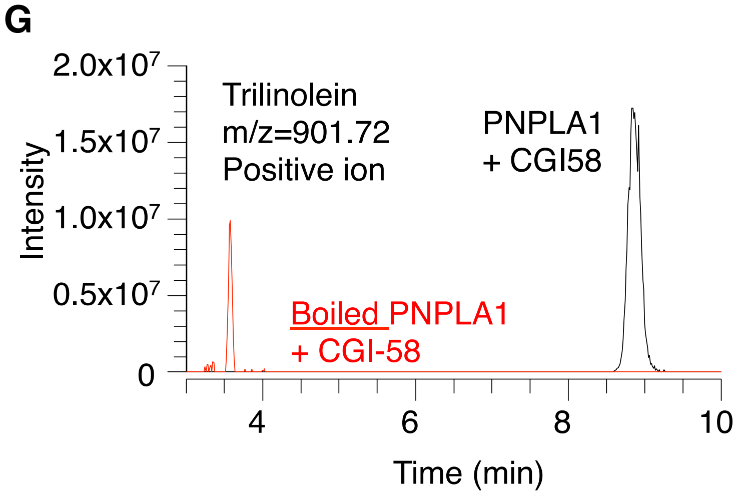

Supplemental Figure 1. Transformations of dilinolein by PNPLA1$_{trun}$/CGI-58 analyzed by LC-MS. **A.** Overlay of LC/MS chromatograms of authentic standards. **B.** Overlay of LC/MS chromatograms of reaction products with boiled PNPLA1$_{trun}$ and CGI-58. **C.** Overlay of LC/MS chromatograms of reaction products with intact PNPLA1$_{trun}$ and CGI-58. **D-G.** Overlay of LC/MS chromatograms of the indicated substrate/product from intact versus boiled PNPLA1$_{trun}$ and CGI-58. **B** and **C** are shown normalized to the intensity/mass ratio of the authentic standards.

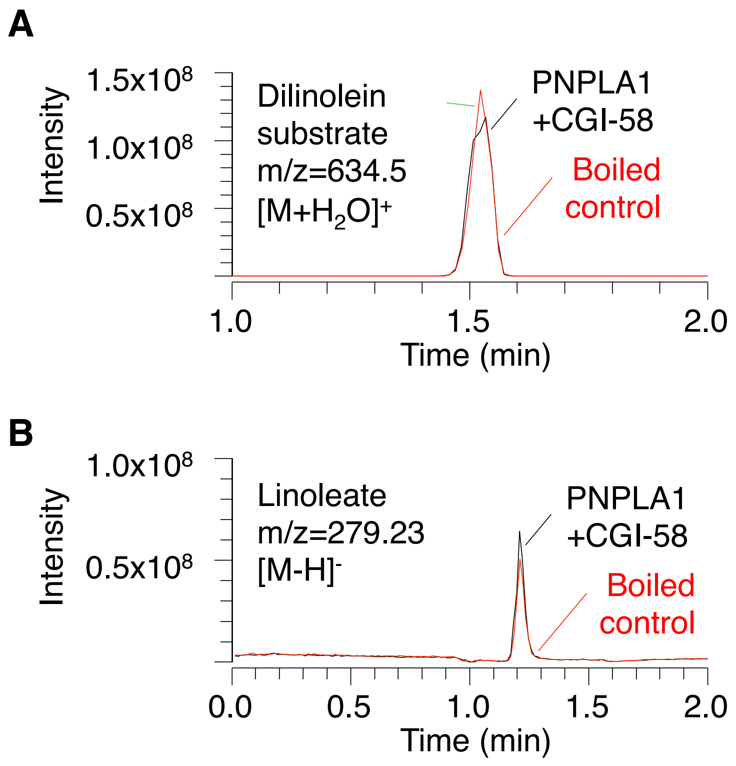

Supplemental Figure 2. Limited activity of full-length PNPLA1$_{FL}$ in transformations of dilinolein. PNPLA1$_{FL}$ was incubated with dilinolein liposomes for 2hr at 37 ˚C and the dilolein substrate remaining (**A**) and linoleic acid formed (**B**) were quantified by LC/MS.

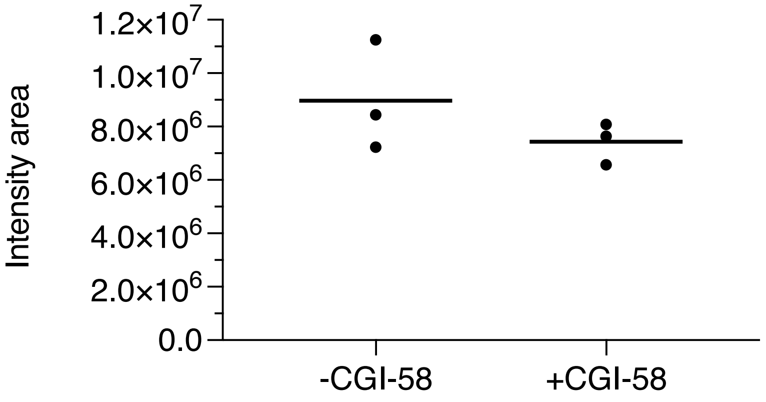

Supplemental Figure 3. Lack of stimulation of PNPLA1$_{trun}$ acylceramide synthesis activity by CGI-58. PNPLA1$_{trun}$ was incubated with dilinolein-OS liposomes +/- CGI-58 for 2 hr at 37 ˚C, and EOS (m/z=1012.96) formed was determined by LC/MS.

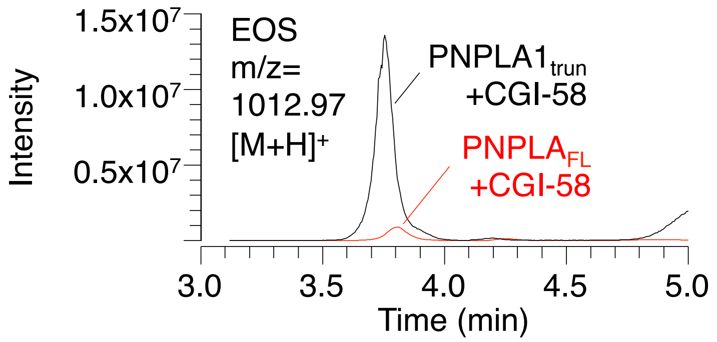

Supplemental Figure 4. Limited activity of full-length $PNPLA1_{FL}$ versus $PNPLA1_{trun}$ in EOS synthesis. $PNPLA1_{FL}$ or $PNPLA1_{trun}$ (matched molar concentrations) were incubated with CGI-58 and dilinolein-OS liposomes for 2 hr at 37 ˚C and EOS formation was determined by LC/MS. A representative overlay of chromatograms is shown.
